# Comparative Transcriptome Profiling Reveals a Potential Role of Type VI Secretion System and Fimbriae in Virulence of Non-O157 Shiga Toxin-Producing *Escherichia coli*

**DOI:** 10.3389/fmicb.2018.01416

**Published:** 2018-06-29

**Authors:** Christina G. Aas, Finn Drabløs, Kjersti Haugum, Jan E. Afset

**Affiliations:** ^1^Department of Clinical and Molecular Medicine, Faculty of Medicine and Health Sciences, Norwegian University of Science and Technology, Trondheim, Norway; ^2^Clinic of Laboratory Medicine, Department of Medical Microbiology, St. Olavs Hospital, Trondheim University Hospital, Trondheim, Norway

**Keywords:** STEC, HUS, virulence, transcriptome, adherence, fimbriae, T6SS

## Abstract

Shiga toxin-producing *Escherichia coli* (STEC) cause both sporadic infections and outbreaks of enteric disease in humans, with symptoms ranging from asymptomatic carriage to severe disease like haemolytic uremic syndrome (HUS). Bacterial virulence factors like subtypes of the Shiga toxin (Stx) and the locus of enterocyte effacement (LEE) pathogenicity island, as well as host factors like young age, are strongly associated with development of HUS. However, these factors alone do not accurately differentiate between strains that cause HUS and those that do not cause severe disease, which is important in the context of diagnosis, treatment, as well as infection control. We have used RNA sequencing to compare transcriptomes of 30 *stx2a* and *eae* positive STEC strains of non-O157 serogroups isolated from children <5 years of age. The strains were from children with HUS (HUS group, *n* = 15), and children with asymptomatic or mild disease (non-HUS group, *n* = 15), either induced with mitomycin C or non-induced, to reveal potential differences in gene expression levels between groups. When the HUS and non-HUS group were compared for differential expression of protein-encoding gene families, 399 of 6,119 gene families were differentially expressed (log2 fold change ≥ 1, FDR < 0.05) in the non-induced condition, whereas only one gene family was differentially expressed in the induced condition. Gene ontology and cluster analysis showed that several fimbrial operons, as well as a putative type VI secretion system (T6SS) were more highly expressed in the HUS group than in the non-HUS group, indicating a role of these in the virulence of STEC strains causing severe disease.

## Introduction

Shiga toxin-producing *Escherichia coli* (STEC) are an important cause of sporadic infections and outbreaks of enteric disease in humans. The symptoms of STEC disease vary, ranging from asymptomatic carriage and diarrhea to hemorrhagic colitis and potentially lethal haemolytic uremic syndrome (HUS). The incidence of STEC infections varies between countries, with lower incidence like 1.81 per 100,000 population in the EU and EEA countries in 2016 (TESSy, 2017) and 2.59 per 100,000 population in the US in 2015 (FoodNet, 2017). Comparatively higher incidence is observed in countries such as Ireland (12.92 per 100,000 population in 2015) and Argentina (10–17 cases per 100,000 children under 5 years old in 2000-2010) (Luis et al., 2014). While O157:H7 was the first STEC serotype to be associated with human disease and remains the most common serotype in many countries, it has become clear that also non-O157 serogroups can cause severe disease. Specifically, the so-called “big six” serogroups, i.e., O26, O45, O103, O111, O121, and O145 are frequently associated with HUS, and in some countries occur more frequently than O157:H7 (TESSy, 2017).

The evolution of pathogenic *E. coli* has likely taken place with multiple parallel acquisitions of important virulence factors, such as pathogenicity islands, plasmids, and prophages. The main virulence factors involved in pathogenesis of STEC are the locus of enterocyte effacement (LEE) pathogenicity island (PAI) and the prophage-encoded Shiga toxin (Stx) (Boerlin et al., 1999; Allison, 2007). LEE encodes a type III secretion system and effectors important for formation of attaching and effacing (A/E) lesions on host cells (McDaniel et al., 1995; Mcdaniel and Kaper, 1997). The Shiga toxin binds to globotriaosylceramide (Gb3) receptors on host cells and upon internalization disrupts protein synthesis leading to cell death (Römer et al., 2007; Schüller et al., 2007). The production and release of Shiga toxin is generally thought to take place as part of the lytic cascade of the phage life cycle and is regulated by the SOS response upon DNA damage. It is thus known to be induced by DNA-damaging agents such as mitomycin C and some antibiotics like fluoroquinolones (Toshima et al., 2007). There are two types (Stx1 and Stx2) and several subtypes of Shiga toxins, where in particular the Stx2a and Stx2d subtypes are known to be more potent and are more frequently associated with development of severe disease than other subtypes (Nataro and Kaper, 1998; Boerlin et al., 1999; Fuller et al., 2011). Other bacterial factors like the O island 122 (OI-122) (Karmali et al., 2003; Wickham et al., 2006), and specific clades or lineages have been reported to be associated with virulence in STEC (Manning et al., 2008; Bono et al., 2012), but none of these factors have been sufficient to reliably identify strains that cause HUS. One of the major problems of such studies is the large genetic heterogeneity of *E. coli* in general and within *E. coli* pathotypes like STEC specifically. Host factors furthermore can contribute to STEC infection. It has been shown that the age of the patient is important both in the risk of acquiring infection and for progression to HUS, i.e., children aged 5 years or less predominantly being affected (Tarr et al., 2005; Tserenpuntsag et al., 2005; Brandal et al., 2015). However, in outbreaks there are commonly observed asymptomatic carriers even in young children, and in the German O104:H4 outbreak in 2011 the majority of cases involved adults (Frank et al., 2011).

Despite extensive knowledge on STEC pathogenesis, current diagnosis of STEC infection is hampered by the inability to reliably distinguish between STEC strains that has the potential to cause HUS from those strains that cause no or mild disease. This is important in the context of diagnosis and treatment, as well as in infection control. We have previously shown, in a comparative genomics study on a large and heterogeneous collection of clinical non-O157 STEC strains, that there were no significant differences in the gene content or genetic variants between high- and low-virulent strains from patients with vs. without HUS, and that different genetic lineages appeared to have independently acquired the virulence factors required for causing severe disease (Haugum et al., 2014). However, it is possible that the observed differences in pathogenic potential lie not with the genetic content of STEC, but with the regulation and expression of virulence genes. In this study, we therefore aimed to investigate if there could be differences in regulation and expression of known or unknown virulence genes that could account for the varying degree of virulence between STEC that had caused HUS and STEC that had caused no or mild disease. Based on the importance of the Shiga toxin prophage in the pathogenicity of STEC, we furthermore decided to investigate differences between HUS and non-HUS strains induced with mitomycin C (induced condition), as well as in a non-induced condition.

## Materials and methods

### Bacterial strains and genome sequences

The *E. coli* strains and respective draft genomes (Haugum et al., 2014; Gabrielsen et al., 2015) used in this study are given in Table 1. Eleven of the strains were isolated from patients with STEC infection in Central Norway in 2001-2014, available from St. Olavs University Hospital (Trondheim, Norway), and 19 were clinical isolates from other parts of the country in the period 2000–2011, available from the Norwegian Institute of Public Health (Oslo, Norway). Of the total 30 strains, 15 had caused HUS and were thus defined as HUS-associated for the purpose of this study, whereas the remaining 15 strains had caused less severe disease and were classified as non-HUS strains. The strains were of different non-O157 serogroups: O26, O121, O103, O145, O177, O111, O86, or non-typeable (termed OUT). All strains shared the main STEC virulence factors associated with severe disease, i.e., Stx2a as well as the LEE pathogenicity island. Furthermore, all strains included in the study were from patients of age 5 years or less.

**Table 1:** Characteristics of clinical and reference strains used in this study.

**Category**	**Strain**	**Accession**	**Phylogroup**	**O-type**	**HUS-associated**	***stx1***	***stx2***	***eae^*a*^***
Clinical strains	FHI63	GCA_000946755	B1	O145	+	–	+	+
	FHI48	GCA_000951915	B1	O121	+	–	+	+
	FHI12	GCA_000937275	B1	O103	+	–	+	+
	FHI24	GCA_000936225	B1	O26	+	–	+	+
	FHI25	GCA_000938995	B1	O145	+	–	+	+
	FHI27	GCA_000951875	B1	O26	+	+	+	+
	FHI79	GCA_000939955	B1	O26	+	–	+	+
	FHI8	GCA_000941395	B1	O86	+	–	+	+
	FHI9	GCA_000939195	B1	O103	+	–	+	+
	FHI83	GCA_000938695	B1	O121	+	–	+	+
	FHI4	GCA_000951835	B1	O26	+	–	+	+
	FHI6	GCA_000941895	B1	O111	+	+	+	+
	FHI7	GCA_000936245	B1	O103	+	–	+	+
	St. Olav164	PVRW00000000	B1	O145	+	–	+	+
	St. Olav176	GCA_000965715	B1	O177	+	–	+	+
	St. Olav104	GCA_000947315	B1	O145	–	–	+	+
	FHI43	GCA_000939755	B1	O121	–	–	+	+
	FHI36	GCA_000753215	B1	O26	–	–	+	+
	FHI66	GCA_000936475	B1	OUT	–	+	+	+
	FHI95	GCA_000753275	E	O145	–	–	+	+
	St. Olav17	GCA_000966935	B1	O121	–	+	+	+
	St. Olav39	GCA_000965545	B1	OUT	–	+	+	+
	St. Olav40	GCA_000965575	B1	OUT	–	+	+	+
	St. Olav63	GCA_000965625	B1	O121	–	–	+	+
	St. Olav157	GCA_000965635	B1	O121	–	–	+	+
	St. Olav172	GCA_000752975	E	O145	–	–	+	+
	St. Olav173	GCA_000965655	E	O145	–	–	+	+
	St. Olav174	GCA_000965665	B1	O26	–	–	+	+
	St. Olav178	GCA_000965555	B1	O177	–	–	+	+
	St. Olav179	GCA_000965705	B1	O26	–	–	+	+
Reference strains	42	FN554766.1	D	O44	–	–	–	–
	REL606	CP000819.1	A	O7	–	–	–	–
	ED1a	CU928162.2	B2	O81	–	–	–	–
	IAI1	CU928160.2	B1	O8	–	–	–	–
	IAI39	CU928164.2	F	O7	–	–	–	–
	CB9615	CP001846.1	E	O55	–	–	–	+
	NRG 857C	CP001855.1	B2	O83	–	–	–	–
	12009	AP010958.1	B1	O103	–	+	+	+
	2011C-3493	CP003289.1	B1	O104	+	–	+	–
	Sakai	BA000007	E	O157	+	+	+	+
	SMS-3-5	CP000970.1	F	O19	–	–	–	–
	K-12 MG1655	U00096.3	A	O16	–	–	–	–
	UMN026	CU928163.2	D	O17	–	–	–	–
	UTI89	CP000243.1	B2	O18	–	–	–	–

a *eae is used as a marker for the LEE pathogenicity island*.

### Ethics

This study was approved by the Regional Committee for Medical and Health Research Ethics (REC) for South East Norway (Ref. 2011/2314). Clinical data (including age and disease severity) required for classification of patients into groups (HUS and non-HUS) were obtained from the Norwegian Surveillance System for Communicable Diseases (MSIS) at the Norwegian Institute of Public Health (NIPH). REK has granted exemption from gaining informed consent, as all data were analyzed anonymously.

### Comparative genomics and core genome phylogeny

The CMG-Biotools package (Vesth et al., 2013) was used for comparative genomics analyses of the 30 STEC genomes and additional 14 *E. coli* reference genomes representing the *E. coli* phylogroups A, B1, B2, D, E, and F (Table 1) (Kaas et al., 2012; Hazen et al., 2013). The core genome was defined as all genes shared across all genomes compared, whereas the accessory genome was defined by subtracting the core genome from the pan genome (i.e., all genes identified across all genomes compared). Minimum 90% identity over minimum 60% alignment length of protein sequences was used as the criterion for defining homologs. A core genome phylogeny, based on alignment of the core genome, was created using FastTree (Price et al., 2009) and visualized using FigTree (Rambaut, 2016).

### Statistical analyses

Fisher's exact test was used to test for differences in presence/absence of accessory protein-encoding gene families between groups, and the Student's *t*-test was used to test for differences in the number of copies of accessory protein-coding gene families between groups. In both cases the Benjamini-Hochberg method was used to control false discovery rate (FDR), with FDR-adjusted *p*-values < 0.05 regarded as statistically significant.

### Culture conditions and induction with mitomycin C

Bacterial cells from a single overnight culture of each strain were washed and diluted in fresh SILAC (stable isotope labeling with amino acids in cell culture) medium optimized for non-auxotrophic *E. coli* (Ping et al., 2013), before incubation at 37°C with agitation until reaching an OD_600_ of ~0.3. The exponential phase culture was then split in two, and one part was induced with mitomycin C at a final concentration of 0.25 μg/ml. Both cultures were subsequently incubated at 37°C with agitation for 1.5 h. Bacterial cells were then spun down and resuspended in 5–10 volumes of RNA later (QIAGEN) before being frozen at −20°C.

### RNA isolation and library preparation

Total RNA was isolated from cell pellets using the Aurum total RNA mini kit (Bio-Rad) according to the manufacturer's instructions, and RNA quality was controlled using the RNA 6000 nano kit and 2100 Bioanalyzer (Agilent). Ribosomal RNA was then removed using Ribo-Zero rRNA Removal Kit for Gram-Negative Bacteria (Epicentre), before library preparation with TruSeq Stranded Total RNA HT Sample Prep Kit (Illumina). Libraries were sequenced with 50 bp single read configuration on a HiSeq 2500 system (Illumina), with an average of 4.06 million reads per sample. Library preparation and sequencing was performed at the Genomics Core Facility (GCF) at the Norwegian University of Science and Technology (NTNU).

### Processing of RNA sequence data

The quality of the RNA sequence data was controlled using FASTQC (Babraham Bioinformatics). Reads were end-to-end aligned, with no mismatches allowed, to the respective draft genomes using Bowtie2 (Langmead and Salzberg, 2012), with an average overall alignment rate of 97.9%. To facilitate comparison of gene expression across strains, all predicted protein-encoding genes from the draft genomes of these strains were clustered into protein-encoding gene families (hereafter termed gene families) using BLASTClust (NCBI), with minimum 90% identity and 60% alignment length of protein sequences as the criterion for defining a family. Reads aligning to each protein-encoding gene family with strand-specific mapping quality above 23 were then counted using HTSeq-count (Anders et al., 2015).

### Differential expression analysis

Differential expression analysis was performed with R (version 3.1.1) and edgeR (version 3.8.5) (Bioconductor) (Robinson et al., 2010). Count data was imported and lowly expressed genes (<1 read per million) filtered away. Normalization factors were calculated using a non-linear Loess model using csaw (Bioconductor) (Lun and Smyth, 2016), and tagwise dispersion was estimated using the weighted likelihood empirical Bayes method. A generalized linear model likelihood ratio test was performed to test for differential expression between groups (HUS vs. non-HUS) and condition (induced vs. non-induced), with FDR-adjusted *p*-values < 0.05 regarded as statistically significant. For further analysis, a log2 fold change threshold > 1 or < −1 was set, i.e., limiting analysis to gene families with >2-fold higher or lower expression between groups.

### Gene ontology and gene cluster analysis

Blast2GO (BioBam) was used for functional annotation based on gene ontology (GO). Differentially expressed genes were defined as belonging to the same gene cluster if they were localized less than 5 ORFs distance from one another. The detected gene clusters were inspected manually in order to identify differentially expressed operons or other genomic regions that were common to strains.

### Identification and characterization of a subset of differentially expressed genes

Based on results from differential gene expression, gene ontology, and gene cluster analysis, differentially expressed gene families that were identified as potentially relevant to the virulence potential of STEC were further investigated. Protein sequences of differentially expressed gene families associated to the GO term for cell adhesion (GO: 0007155) were extracted and queried using blastp (version 2.2.29+) against a set of known STEC adherence factors (fimbriae, pili, curli, adhesins, and autotransporters) from the Virulence Factor DataBase (VFDB), and against the reference genomes of *E. coli* K12 (CP014225) and *E. coli* O111:H- 11128 (NC_013364) for identification. Protein sequences of all differentially expressed gene families were furthermore queried against the SecReT6 database (Li et al., 2015) for identification of type VI secretion system components, effectors and immunity proteins.

### Data availability

The datasets generated for this study are available from the NCBI's Gene Expression Omnibus, GEO Series accession number GSE112430.

## Results

### Comparative genomic analyses of HUS and non-HUS strains

Comparative genomic analyses of the 30 clinical STEC strains included in this study as well as 14 reference strains representing the *E. coli* phylogroups A, B1, B2, D, E, and F (Table 1) revealed considerable heterogeneity in the genetic content of the clinical strains, which is often the case with pathogenic *E. coli*. The core genome was found to consist of 2,847 shared gene families, whereas the pan genome comprised a total of 11,769 gene families.

**Figure 1:**
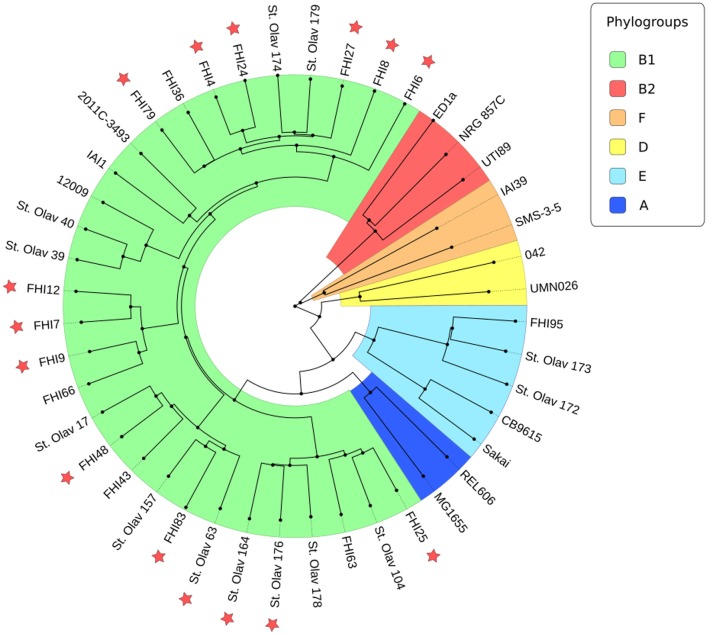
Core genome phylogeny of clinical and reference strains. STEC strains associated with HUS are indicated by red stars, and phylogroups are colored according to the legend.

In order to establish if there were genetic differences that could account for the varying degree of virulence between the STEC strains in this subset, a phylogenetic tree was constructed based on a core genome alignment. The core genome phylogeny displayed in Figure 1 showed that a majority of the 30 non-O157 STEC strains belonged to the B1 phylogroup, and that the strains in general clustered in accordance to serogroup/serotype. However, there did not appear to be a clear correlation between the core genome and degree of virulence, as most HUS strains clustered together with non-HUS strains. Potential differences between HUS and non-HUS strains in the accessory genome was investigated using Fisher's exact test for presence/absence of accessory gene families between groups, and the students *T*-test for the number of copies of accessory gene families between groups. Among 136 gene families found to be differentially distributed (presence/absence) between the HUS group and the non-HUS group by uncorrected *p*-values (*p* < 0.05) (Table S1), 57% were annotated as hypothetical proteins with unknown function. When corrected for multiple hypothesis testing none of these were statistically significant (FDR < 0.05). Similarly, there were 343 gene families that showed differences in copy number between the HUS group and the non-HUS group by uncorrected *p*-values (*p* < 0.05), but when corrected for multiple hypothesis testing only two were statistically significant (FDR < 0.05) (Table S2). These two gene families were annotated as hypothetical proteins with unknown function.

### The STEC transcriptome and differential expression analysis

**Figure 2:**
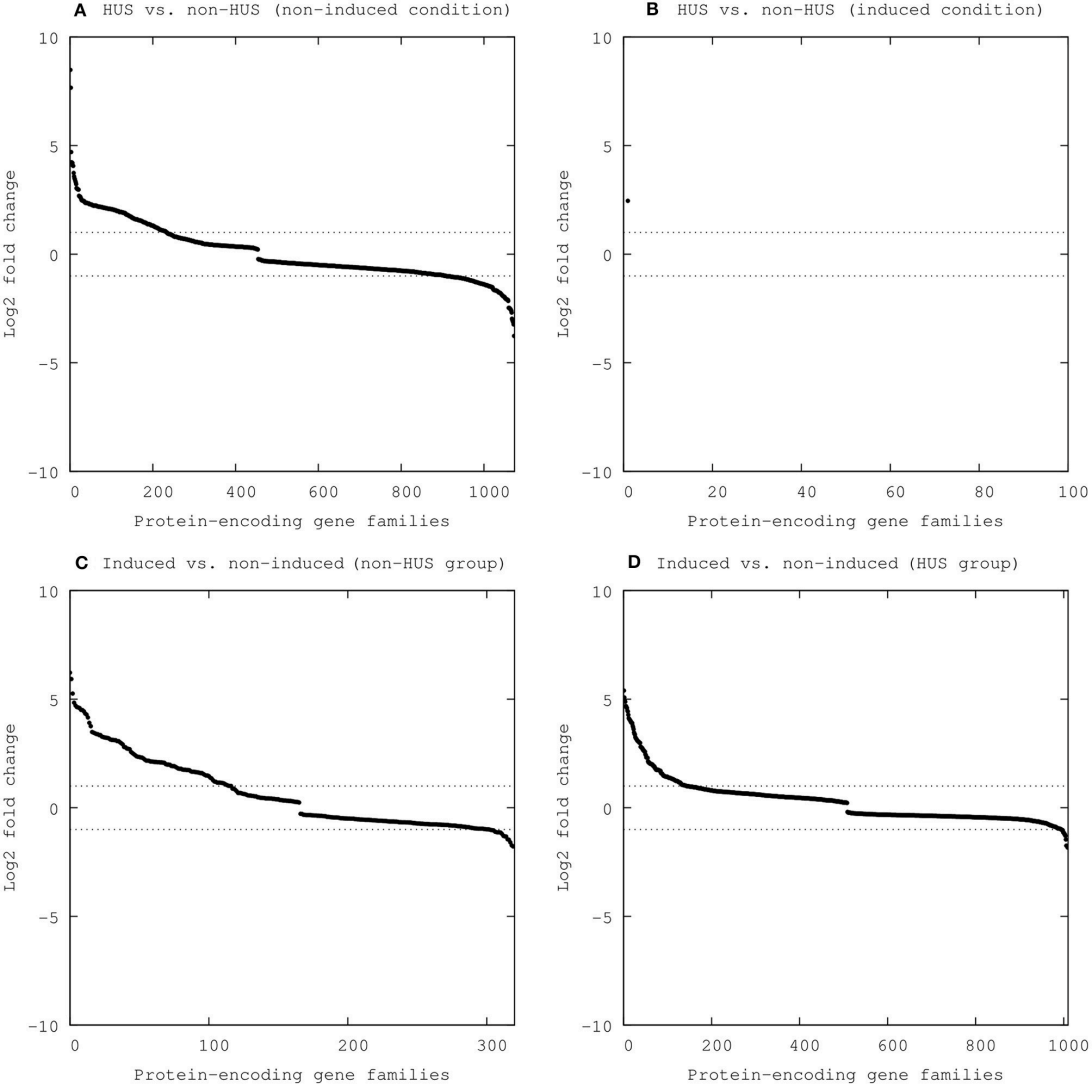
Differentially expressed protein-encoding gene families between groups (HUS vs. non-HUS) and conditions (induced vs. non-induced). Statistically significant results (FDR < 0.05) are included, with expression shown as log2 fold change (y-axis) for each gene family (x-axis) **(A)** HUS vs. non-HUS (non-induced conditions), **(B)** HUS vs. non-HUS (induced conditions), **(C)** Induced vs. non-induced (non-HUS group), and **(D)** Induced vs. non-induced (HUS group).

We identified 5,719 gene families as being expressed in one or more of the 30 STEC strains either in the induced or the non-induced condition, whereas 3,166 of these gene families were expressed in all strains in all conditions. We used a generalized linear model likelihood ratio test to test for differential expression between groups (HUS vs. non-HUS), and conditions (induced vs. non-induced) with an adjusted *p*-value cutoff of 0.05. The results from differential expression analyses are shown in Figure 2, as log2 fold change (FC) for each protein-coding gene family.

### Differential expression between the HUS and non-HUS group

The results for differential gene expression between the HUS and non-HUS group in both the induced and the non-induced condition are shown in Figure 2 and summarized in Table 2. Two hundred and thirty-five gene families were more highly expressed in the HUS group than in the non-HUS group. Among these, one gene family was unique for the induced condition while 234 gene families were more highly expressed in the non-induced condition. One hundred and sixty-five gene families were expressed at a lower level in the HUS group than in non-HUS group, all unique to the non-induced condition (Table S3). There were no statistically significant differences in expression levels of 5,719 gene families.

**Table 2:** Differentially expressed protein-encoding gene families.

**Compared groups**		**Lower**	**Unchanged**	**Higher**
HUS vs. non-HUS	Unique for induced condition	0	399	1
	Unique for non-induced condition	165	1	234
	Common for both conditions	0	5319	0
	Total	165	5719	235
Induced vs. non-induced	Unique for HUS-group	10	39	53
	Unique for non-HUS group	12	63	27
	Common for both groups	5	5522	90
	Total	27	5624	170

*Differentially expressed gene families with 2-fold change or higher between groups (HUS vs. non-HUS) and conditions (induced vs. non-induced) are included*.

**Figure 3:**
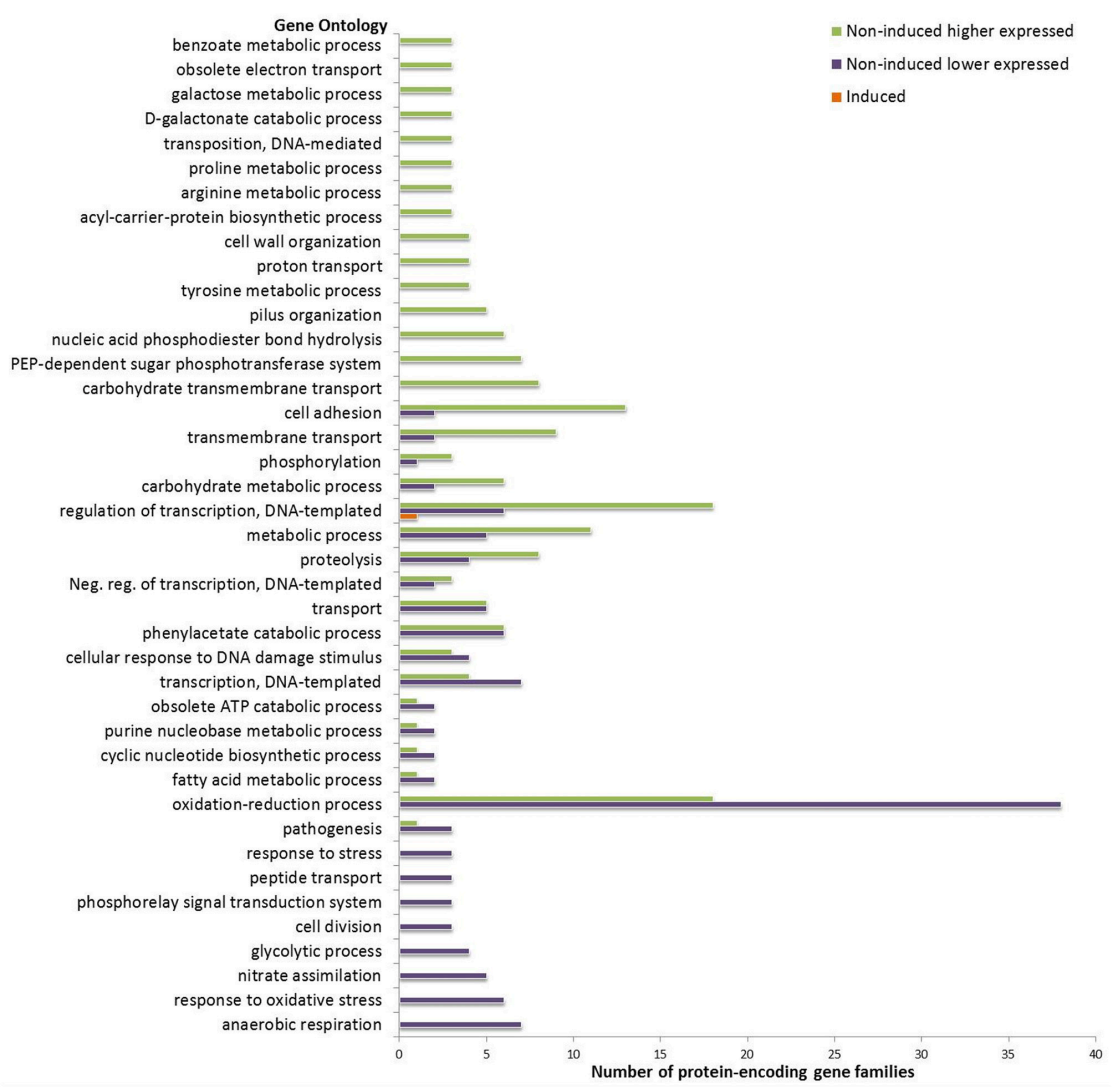
Gene ontologies of differentially expressed protein-encoding gene families in the HUS vs. non-HUS group. Biological process gene ontologies (y-axis) where the number of gene families assigned (x-axis) is higher than one are included. Subgroups (induced and non-induced condition) are colored according to the legend.

Gene ontology analysis provided ontology terms for a majority (overall 73.3%) of the differentially expressed gene families in the HUS group relative to the non-HUS group. Among the differentially expressed gene families in the non-induced condition, the biological process gene ontologies for response to (oxidative) stress and anaerobic respiration were lower expressed in the HUS group, whereas ontologies for diverse metabolic processes involving amino acids, carbohydrates, and nucleic acids were higher expressed in this group (Figure 3). Some GO terms were associated with both higher and lower expressed gene families in the HUS group, including GOs for oxidation-reduction process, regulation of transcription (negative regulation of transcription and DNA-templated regulation of transcription), and several metabolic processes. Of note, the GO terms for cell adhesion and pilus organization appeared to be more highly expressed in the HUS group.

A closer inspection of the differentially expressed gene clusters related to the GO terms for cell adhesion and pilus organization revealed that among the more highly expressed genes in the HUS group were genes located in 8 chaperone-usher (CU) fimbriae operons (Table 3). These operons encode among others well-characterized fimbriae like type 1 and F9 fimbriae. Type 1 fimbriae are known to mediate binding to α-D-mannosylated receptors which are abundant in the bladder (Korea et al., 2010), while F9 fimbriae have been shown to mediate biofilm formation and bind to terminal Galβ1-3GlcNAc structures found in the human urinary tract and kidney and part of the lactotetraosylceramide glycosphingolipid receptor present in human gastric epithelium (Wurpel et al., 2014). Furthermore, this includes the fimbrial operons encoding Ybg and Yfc fimbriae, which have been shown to increase adherence to bladder cells (Korea et al., 2010). Additionally, a number of less characterized fimbrial operons, encoding Yra, CS1-like, and Yqi-like fimbriae (Wurpel et al., 2013) displayed higher expression in the HUS group.

**Table 3:** Identity and function of a selection of differentially expressed protein-encoding gene families between the HUS and non-HUS group.

**Gene family**	**Log2 FC**	**Gene Ontology**	**Annotation**	**ID**	**Putative function**	**Present in no. strains**	
						**HUS**	**non-HUS**
4021	1.56	Nucleic acid phosphodiester bond hydrolysis	Secreted protein Hcp	*hcp*	Type VI secretion system	15	12
3905	2.37	NA	IcmF-related protein	*IcmF*	Type VI secretion system	15	12
3990	2.05	NA	Type VI secretion protein VasI	–	Type VI secretion system	15	12
3907	2.37	Proteolysis	ClpB protein	*clp*	Type VI secretion system	15	12
58	2.99	NA	VgrG protein	*vgrG*	Type VI secretion system	15	12
99	3.00	Cellular response to phosphate starvation, dephosphorylation, cellular response to anoxia	core protein	–	Rhs protein	15	12
23	3.04	Transposition, DNA-mediated	Mobile element protein	–	–	15	12
3790	−2.97	Pathogenesis	Probable glutamate/gamma-aminobutyrate antiporter	–	–	13	15
3860	1.31	Cell adhesion	type 1 fimbrae adaptor subunit FimF	*ydeS*	F9 fimbriae	15	13
215	1.39	Oxidation-reduction process	Mobile element protein	Z4324	OI-122	15	14
611	1.19	Oxidation-reduction process	Enterotoxin	*espL*	OI-122	15	14
4065	2.48	NA	FIG00638171: hypothetical protein	EDL933_4193	OI-122	15	12
3569	1.25	NA	FIG00638856: hypothetical protein	*nleB*	OI-122	15	14
2325	1.08	Oxidation-reduction process	Unknown function	*nleE*	OI-122	15	14
3869	−1.63	Oxidation-reduction process, response to stress	Type III secretion chaperone protein for YopD (SycD)	–	–	13	15
3865	1.32	NA	PilT	–	–	15	13
4042	1.76	NA	FIG01200701: hypothetical protein	*ygiC*	Yqi-like fimbriae	15	12
4014	2.15	Cell adhesion	Uncharacterized fimbrial-like protein ygiL precursor	*YgiL*	Yqi-like fimbriae	15	12
3912	2.36	Transport	Uncharacterized outer membrane usher protein yqiG precursor	*ygiG*	Yqi-like fimbriae	15	12
3908	2.98	NA	Alpha-fimbriae usher protein	–	Cs1-like fimbriae	15	12
4017	1.94	NA	Alpha-fimbriae major subunit	–	Cs1-like fimbriae	15	12
4010	1.99	Cell adhesion	Putative fimbrial-like protein	*yraH*	Yra fimbriae	15	12
3997	1.93	Pilus organization	Chaperone protein fimC precursor	*yraI*	Yra fimbriae	15	12
3911	2.45	Transport	type 1 fimbriae anchoring protein FimD	*yraJ*	Yra fimbriae	15	12
2526	−1.16	Oxidation-reduction process, response to oxidative stress, negative regulation of transcription, DNA-templated	Flavoprotein wrbA	–	Insertion site of Stx phage	15	15
62	1.05	NA	putative virulence protein	*insQ*	IS609 transposase B	15	14
90	2.08	Transposition, DNA-mediated	Mobile element protein	*insP*	IS609 transposase A	15	13
2890	−1.14	Cell adhesion	Universal stress protein F	*uspF*	Insertion site of phage	15	15
3840	1.18	NA	HTH-type transcriptional regulator mlrA	*mlrA*	Insertion site of Stx phage	15	13
4012	1.90	Cell adhesion	Major fimbrial subunit StfA	*YfcV*	Yfc fimbriae	15	12
3849	1.55	Regulation of transcription, DNA-templated, transcription, DNA-templated, DNA integration, DNA recombination	type 1 fimbriae regulatory protein FimE	*fimE*	type-1-fimbriae	15	13
3856	2.29	Cell adhesion	type 1 fimbriae major subunit FimA	*fimA*	type-1-fimbriae	15	13
3858	1.68	Cell adhesion	type 1 fimbriae protein FimI2C unknown function	*fimI*	type-1-fimbriae	15	13
3841	1.72	Pilus organization	chaperone FimC	*fimC*	type-1-fimbriae	15	13
1	1.13	NA	Mobile element protein	–	type-1-fimbriae	15	14
3859	1.62	Cell adhesion	type 1 fimbrae adaptor subunit FimF	*fimF*	type-1-fimbriae	15	13
3863	1.40	Cell adhesion, pilus organization	type 1 fimbrae adaptor subunit FimG	*fimG*	type-1-fimbriae	15	13
3822	1.67	Cell adhesion	mannose-specific adhesin FimH	*fimH*	type-1-fimbriae	15	13
4106	−2.51	Cell adhesion	FIG01069793: hypothetical protein	–	–	11	15
3624	−1.04	Pathogenesis	Methionine aminopeptidase (EC 3.4.11.18)	–	–	14	15
4040	2.12	Nucleic acid phosphodiester bond hydrolysis	CRISPR-associated protein2C Cas2	*cas2*	CRISPR/Cas	15	12
3964	2.24	nucleic acid phosphodiester bond hydrolysis, defense response to virus, maintenance of CRISPR repeat elements	CRISPR-associated protein Cas1	*cas1*	CRISPR/Cas	15	12
4003	2.18	NA	CRISPR-associated protein2C Cse3 family	*cas6*	CRISPR/Cas	15	12
3989	1.94	Defense response to virus	CRISPR-associated protein2C CT1976	*hyp*	CRISPR/Cas	15	12
3950	2.09	NA	CRISPR-associated protein2C Cse4 family	*cse4*	CRISPR/Cas	15	12
4172	3.31	Cell adhesion	Fimbriae-like adhesin SfmA	–	LPFB1 fimbriae	15	10
4168	3.45	Pilus organization	Putative fimbrial chaperone protein	–	LPFB1 fimbriae	15	10
4158	4.21	Transport	type 1 fimbriae anchoring protein FimD	–	LPFB1 fimbriae	15	10
4163	3.59	Cell adhesion	Putative fimbrial protein	–	LPFB1 fimbriae	15	10
3639	−1.07	Pathogenesis	Phosphate transport system regulatory protein PhoU	–	–	14	15
3451	1.52	Pathogenesis	FIG00638466: hypothetical protein	–	–	15	14
3588	1.06	Chemotaxis, archaeal, or bacterial-type flagellum-dependent cell motility	Flagellar motor rotation protein MotB	–	–	15	14
4011	1.61	Cell adhesion	Uncharacterized fimbrial-like protein ygiL precursor	*ybgD*	Ybg fimbriae	15	12
3913	2.34	Transport	Uncharacterized outer membrane usher protein yqiG precursor	*ybgQ*	Ybg fimbriae	15	12
3991	1.98	Pilus organization	Periplasmic fimbrial chaperone	*ybgP*	Ybg fimbriae	15	12
3953	2.03	Cell adhesion	FIG00637862: hypothetical protein	*ybgO*	Ybg fimbriae	15	12
23	3.04	Transposition, DNA-mediated	Mobile element protein	*ybfL*(partial)	DDE domain transposase family	15	12
4242	4.23	NA	orf2C hypothetical protein	*ybfC*	putative secreted protein	15	9
68	8.49	NA	core protein	*rhsC*	Rhs protein	15	5

*Differential expression is shown as log2 fold change. Identification and putative functions are based on gene ontology, annotation and blast against E. coli reference genomes and virulence factor databases*.

Another CU operon, which was found in 10 of the non-HUS strains and 15 of the HUS strains, had 4 ORFs with 9.9 to 12-fold increased expression in the HUS group. The CU operon was identified in ~50% of phylogroup B1 strains, with 99–100% nucleotide identity, based on blast against 106 non-O157 STEC strains that we have previously sequenced (Haugum et al., 2014; Gabrielsen et al., 2015) and 14 reference genomes. The encoded proteins display some similarity (42–63% identity) to long polar fimbriae 2 (LPF2) proteins of *E. coli* O157:H7 Sakai (Figure S1) and is found in the same locus (between *glmS* and *pstS* in O island 154). For clarity, we hereafter refer to the operon as *lpf*_*B*1_. In contrast to the *lpf2* operon in O157:H7 Sakai and other phylogroup E strains, *lpf*_*B*1_ encodes only 4 proteins: LpfA (adhesin), LpfB (chaperone), LpfC (usher), and LpfD (adhesion) (Table 3). A short fifth ORF, which most likely is a pseudogene and not differentially expressed between the HUS and non-HUS groups, is found in only 4 of our clinical strains. The *lpf*_*B*1_ encoded fimbriae have previously been identified in other non-O157 strains and shown to be important for adherence to epithelial cells in EHEC O113:H21 (Doughty et al., 2002) as well as in serotype O78 ExPEC strains (Ideses et al., 2005). In this study, homologous *lpf* operons were also identified in phylogroups D and F, with 89% nucleotide identity, but not in strains from phylogroup A and B2. This could suggest a more recent horizontal transfer of this gene cluster. We also found that the presence of the *lpf*_*B*1_ operon is significantly associated with *stx2a* (*p* = 0.01) when tested in a larger collection of 95 non-O157 STEC strains (Haugum et al., 2014) using Fisher's exact test.

Most of the CU fimbrial operons have been shown to be regulated by the Histone-like nucleoid structuring protein (HNS) which is a global negative regulator that regulates 5% of all *E. coli* genes (Hommais et al., 2001), with a preference for horizontally acquired genes (Oshima et al., 2006). In this study, we found a small (fold change 0.77) but not statistically significant decreased expression of HNS in the HUS group compared to the non-HUS group (uncorrected *p*-value < 0.05, FDR-adjusted *p*-value 0.11). Nor were other investigated global regulators (CRP, FIS, IHF), or regulators connected to HNS (StpA, Hha, Ydgt) significantly differentially expressed between the HUS and non-HUS group.

A putative type VI secretion system (T6SS) gene cluster present in the phylogroup B1 strains (15 HUS strains and 12 non-HUS strains), encoding proteins with 99–100% identity to characterized T6SS components, was found to be 3 to 8-fold more highly expressed in the HUS group than in the non-HUS group (Table 3). The differentially expressed genes encoded structural and/or effector proteins of T6SS, including Hcp (hemolysin-coregulated protein), IcmF (intracellular multiplication protein F), Clp, and VgrG (valine glycine repeat) (Ma et al., 2012; Zhou et al., 2012; Ho et al., 2014). The T6SS gene cluster, which is highly conserved in pathogenic *E. coli* strains, has previously been shown to be functional and to be repressed by the global regulator HNS (Wan et al., 2017).

Two of the most highly differentially expressed gene families in this study include large (~1400 amino acids) rearrangement hotspot (Rhs) proteins or core proteins (Table 3). One of these gene families, found in all phylogroup B1 strains, was located adjacent to the putative T6SS gene cluster and was expressed 8-fold higher in the HUS than in the non-HUS group. The other family was found more frequently in HUS strains (*n* = 15) than in non-HUS strains (*n* = 5) and displayed 360-fold higher expression in the HUS group. Another 30 gene families encoding core/Rhs proteins were however not differentially expressed.

Of note is also the finding that several genes of the OI-122 pathogenicity island were found to be more highly expressed in the HUS group relative to the non-HUS group in the non-induced condition (Table 3). These genes encode the type III secretion system effectors NleE, NleB, and Sen (Shigella enterotoxin) from module 2 of this PAI, which are known to be involved in inhibition of the host cell inflammatory response during formation of attaching and effacing lesions (Newton et al., 2010).

### Transcriptional responses to induction

In the induced condition, a total of 170 gene families were found to be more highly expressed and 27 were expressed at lower levels compared to the non-induced condition (Figure 2, Table 2 and Table S3). Among gene families more highly expressed, 90 were common to both the HUS and non-HUS group. Of gene families expressed at lower levels, 12 were unique to the non-HUS group and 10 unique to the HUS group. There was no significant difference in expression level between the induced and the non-induced condition for 5,624 gene families.

Gene ontology analysis of the differentially expressed gene families in the induced condition provided one or more GO terms for 46.7% of these, whereas the rest of the gene families were not assigned to any GO term. A majority of the unassigned gene families were annotated as phage-related (28.4%) or hypothetical proteins (41.1%).

**Figure 4:**
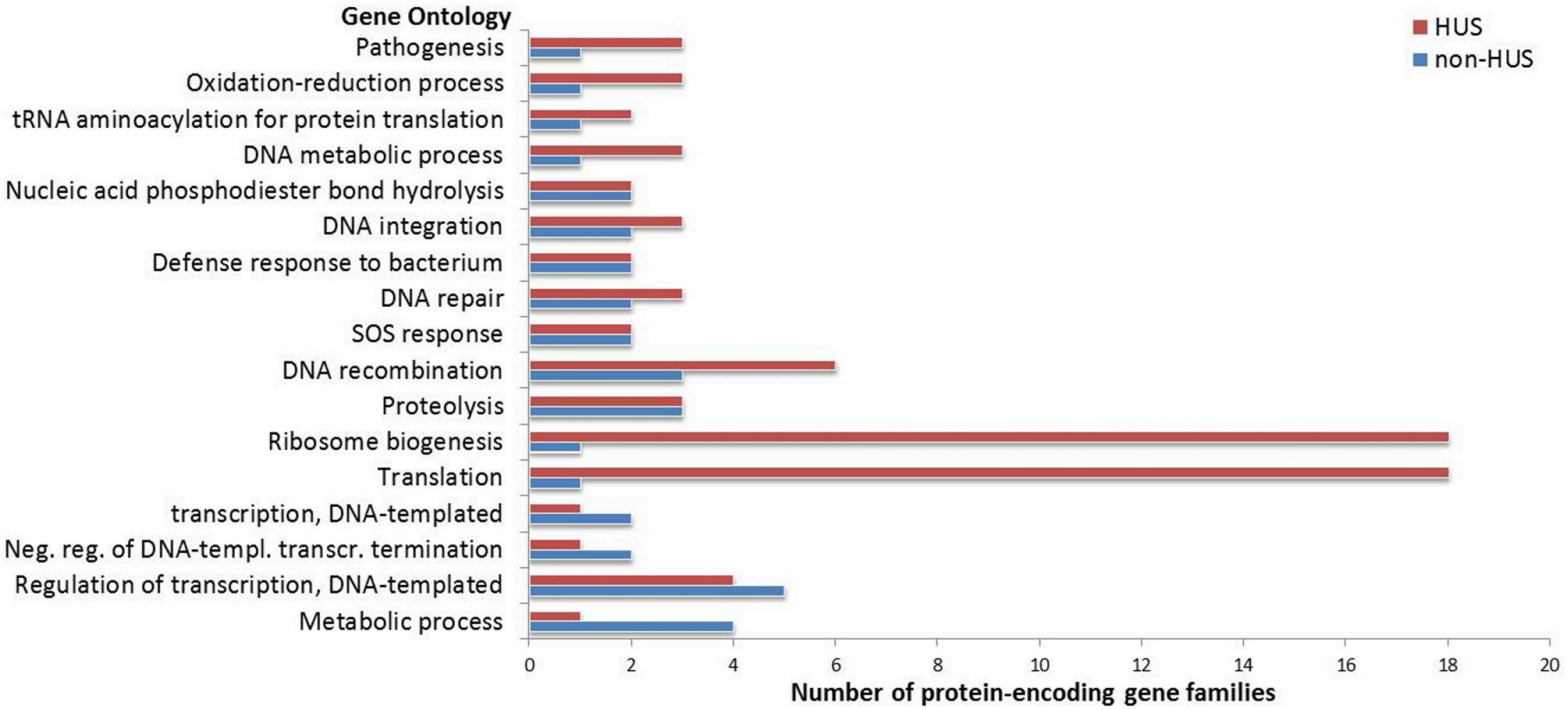
Gene ontologies of differentially expressed protein-encoding gene families in the induced vs. non-induced condition. Biological process gene ontologies (y-axis) where the number of gene families assigned (x-axis) is higher than one are included. Subgroups (HUS and non-HUS) are colored according to the legend.

Of the more highly expressed gene families in the induced condition, the biological process gene ontologies for regulation of transcription (DNA-templated), SOS response, DNA recombination, repair, and integration as well as proteolysis were common to both groups (Figure 4). This includes genes known to be involved in the SOS response, like *lexA, recA, recN, dinI, yebG*, and *sulA*. Analysis of differentially expressed gene clusters furthermore revealed that a majority of the genes encoded by the Shiga toxin prophage were induced in one or both groups. This includes well-known prophage genes encoding proteins such as integrase and excisionase (Int, Xis), regulators involved in lysis (Cro and Q antiterminator protein), replication and recombination proteins (recombinases, primase, helicase, ATPase, methyltransferases), the Shiga toxin (Stx2A and Stx2B), cell division inhibition protein (Kil), lysis proteins (S and R), and packaging and structural proteins (DNA packaging proteins, minor tail proteins, and portal protein). Although short reads and highly conserved prophage gene families makes it difficult to distinguish between expression originating from closely related prophages, it appears that a few other prophages also responded to mitomycin C treatment. A gene cluster encoding genes homologous to *yfdSRQP* encoded by the cryptic prophage CPS-53 of *E. coli* K12 were more highly expressed in the induced condition, whereas genes involved in regulation and recombination from a 933W-like phage were also induced. Furthermore, the *groES* and *groEL* genes of the groEL-groES chaperonin complex, which is known to be involved in lambda, T4 and T5 phage assembly (Tilly et al., 1981) in addition to its essential role in protein folding, were more highly expressed in the induced condition.

Some effects of induction on expression levels were significant only within groups. Interestingly, the LEE IV operon genes *espB, espD*, and *eae* were expressed at lower levels in the induced condition within the HUS group, whereas several ribosomal protein gene clusters were more highly expressed in the induced condition also within the HUS group.

Genes of the GAD acid fitness island (*gadABC*) were expressed at lower levels in the induced condition than in the non-induced condition. A previous study has implicated Shiga toxin prophage carriage in upregulation of this operon (Veses-Garcia et al., 2015), and the results of our study further supports the existence of a regulatory link between Shiga toxin prophages and the gad operon.

## Discussion

Comparative genomic analyses have previously not been able to establish any correlation between the degree of virulence and the genetic content of STEC strains, which was also shown in this study (Figure 1 and Table S1). To investigate if the differences in pathogenic potential could be associated with regulation and expression of virulence genes, we therefore used RNA sequencing to compare gene expression between STEC that cause HUS and STEC that cause no or mild disease. We identified several genes that were differentially expressed between HUS and non-HUS strains, findings which could thus contribute to a better understanding of STEC virulence. Better classification of the virulence potential of STEC-strains would be of considerable importance for more accurate diagnosis, treatment and infection control of STEC infection.

A large number of genes were differentially expressed between STEC of the HUS and the non-HUS groups in the non-induced condition, while perhaps surprisingly, there were few differences in expression between the groups in the induced condition (Figure 2 and Table 2). Among the most notable findings were a number of fimbrial operons, which were more highly expressed in the HUS group compared to non-HUS group. These include both well-known fimbriae (type 1 and F9 fimbriae) and fimbrial operons which have not been extensively studied (Table 3), but which could be of importance for attachment and tissue tropism of STEC. These results not only provide evidence for expression of these operons in wild-type strains, but could also indicate a role of these operons in the increased virulence of the HUS group. Another interesting finding was a putative type VI secretion system gene cluster which was more highly expressed in the HUS group (Table 3). T6SS is one of the more recently discovered secretion systems in Gram-negative bacteria, and has many unknown effectors. It has however been shown to contribute to pathogenicity, as well as to have a role in host immunomodulation and interbacterial interactions (Ho et al., 2014). Specifically, it has been shown that several enteric pathogens use the T6SS to attack members of the host commensal microbiota, thus facilitating colonization of the gut (Sana et al., 2016; Anderson et al., 2017; Zhao et al., 2018), and that commensals in turn can antagonize pathogens using the T6SS, providing the host with colonization resistance (Chatzidaki-Livanis et al., 2016; Casterline et al., 2017). It has previously also been demonstrated that T6SS attenuates virulence of a STEC strain in an animal model (Wan et al., 2017), and that the T6SS effectors Hcp1 and Hcp2 contribute to binding and invasion of human brain microvascular endothelial cells of the meningitis-associated *E. coli* K1 strain (Zhou et al., 2012). Our findings indicate that T6SS may also have a role in the increased pathogenicity of STEC causing HUS, possibly by aiding in colonization of the gut and thus increasing the bacterial load. A third observation was that of very high expression of two Rhs protein families (8-fold and 360-fold higher expression) in the HUS group (Table 3). Due to the short reads and repetitive nature of these genes, the true origin (i.e., specific ORFs) of the reads can be uncertain. However, it is clear that these Rhs genes were much more highly expressed in the HUS than in the non-HUS group under these conditions. The functions of Rhs elements are not well-understood, but their features are often suggestive of cell-surface ligand binding functions, and they have been shown to mediate intercellular competition in other Gram-negative bacteria (Hill et al., 1994; Koskiniemi et al., 2013). Notably, several Rhs proteins are known to be effectors of the T6SS, where the Rhs repeat domain forms a structure that encloses and protects a folded enzymatically active effector domain (Alteri and Mobley, 2016). Although the putative functions of the differentially expressed Rhs proteins are unknown, our results could indicate a hitherto unknown role of Rhs effectors in the virulence of STEC causing HUS. Interestingly, although there was high strain-to-strain variation, the Shiga toxin or other Stx prophage genes were not significantly differentially expressed between the HUS and non-HUS group, neither in the induced nor in the non-induced condition. This indicates that increased pathogenic potential is not simply a matter of increased Shiga toxin production under these conditions, which has been suggested previously (Ogura et al., 2015). Nor were Core LEE genes differentially expressed between the groups. However, our findings do show higher expression of some non-LEE effectors from the OI-122 in the HUS group. The higher expression of these effectors could potentially inhibit the host inflammatory response more efficiently and thus contribute to the higher pathogenic potential of the HUS strains.

There were few differences in expression between the HUS and non-HUS groups in the induced condition (Figure 2 and Table 2). Of the 170 upregulated genes, 90 were common to both the HUS and non-HUS group. These include well-known genes involved in the SOS response and from the Shiga toxin prophage. Furthermore, we observed that other prophages in the *E. coli* genome responded to mitomycin C induction, including an operon from the cryptic prophage CPS-53 of *E. coli* K12 and a 933W-like phage. However, GO analysis provided annotation for only 53.3% of the differentially expressed genes (Figure 4), meaning that there are still a large number of genes that are expressed upon induction that do not have any known function. A possible problem with the comparison between the induced and non-induced groups is that although the growth phase of cultures were synchronized at the time of induction, after 1.5 h incubation the non-induced cells would have continued dividing and reached early stationary phase while the induced cells would have stopped dividing and may have eventually lysed. This could have influenced results of this comparison, in that we observed not only the effects on induction itself, but also the effects of differences in growth phase at the time of sampling.

To adjust for the variability caused by different Stx subtypes and the presence or absence of LEE, we only included strains with the Stx2a subtype and the LEE pathogenicity island in this study (Table 1). We furthermore chose to focus on non-O157 serogroups due to the large burden of STEC infections associated with non-O157 serotypes in several European countries, but also increasingly in the US (FoodNet, 2017; TESSy, 2017). By excluding O157 strains, we could also gain a better understanding of virulence traits that are important for pathogenicity, unbiased by the dominating role of O157 STEC. All strains included in the study were from patients of age 5 years or less, to reduce the influence of age as a risk factor for severe STEC disease. Information on other host and environmental factors, which could potentially affect the susceptibility to and severity of disease, were not available and could therefore not be adjusted for in this study. Such factors include among others the infectious dose, comorbidities, genetic background, and immune response of the host.

In this study cells were grown until the exponential phase in SILAC medium optimized for non-auxotrophic *E. coli* (Ping et al., 2013). The medium was chosen for its applicability to future proteomics studies. This is a chemically defined medium containing glucose, M9 salts, amino acids, nucleobases and vitamins. Although not a minimal medium per definition, growth of the clinical STEC strains in this medium was slow (approximate doubling time of 43 min) and did not reach the high densities observed in rich media. The growth conditions in SILAC medium compared to that of the lower human intestine are clearly not similar. However, performing the growth experiments in a nutrient-limiting medium rather than in a rich medium could in this aspect better reflect the conditions in the lower intestine, where *E. coli* competes with the rest of the microbiota for nutrients.

Total RNA sequencing is a powerful, open and relatively unbiased approach, which allows for accurate identification and quantification of the highly dynamic bacterial transcriptome. Importantly however, the careful design of experimental conditions may have considerable impact on the interpretation of results. A major limitation of this study, and similar studies of gene expression in STEC, is the lack of good model systems which can mimic both the initial phases of the STEC infection, i.e., adherence and colonization of the human intestinal epithelium, as well as the systemic and local effects of Shiga toxins. However, although one cannot readily extrapolate from bacterial cell culture to *in vivo* effects, studies like this may provide important early findings that can subsequently be tested in relevant model systems.

In conclusion, we have used total RNA sequencing to identify global differences in gene expression between high- and low-virulent STEC, and identified a number of genes and operons which may be of importance for STEC pathogenesis, some of which have not been previously well-characterized. The study thus provides new insights and opportunities for functional studies, which may lead to more accurate diagnosis and treatment, and more appropriate infection control measures for infection with high and low risk STEC strains.

## Author contributions

CA, JA, FD, and KH conceived and designed the experiments. CA performed the experiments. CG analyzed the data. CA, JA, FD, and KH contributed reagents, materials, analysis tools. CA, KH, FD, and JA contributed to the writing of the manuscript.

### Conflict of interest statement

The authors declare that the research was conducted in the absence of any commercial or financial relationships that could be construed as a potential conflict of interest.
