# Supplementary figures and images for: Comparative Transcriptome Profiling Reveals a Potential Role of Type VI Secretion System and Fimbriae in Virulence of Non-O157 Shiga Toxin-Producing Escherichia coli
Source: Front Microbiol. 2018 Jun 29;9:1416. doi: 10.3389/fmicb.2018.01416 (PMC6033998; doi:10.3389/fmicb.2018.01416)

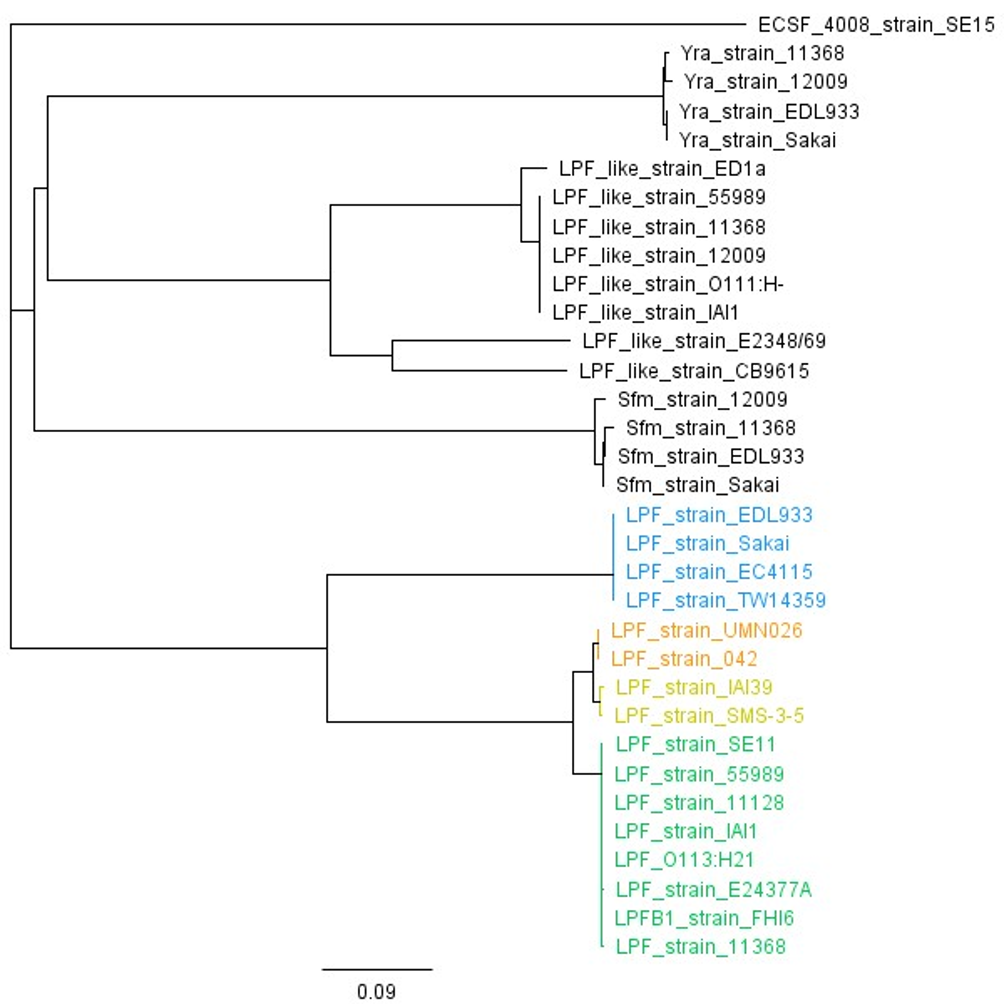

Supplement: Supplementary file 4 [file Image_1.tiff]
